# A Simple Ophthalmoscope

**Published:** 1864-11

**Authors:** J. Z. Laurence


### A SIMPLE OPHTHALMOSCOPE.

Sir:—I find that if a convex lens of about two inches focus be placed in close apposition with a concave one of about nine inches focus, and this combination be held before the patient's eye at the distance the object-lens of an ordinary ophthalmoscope usually is, it forms an ophthalmoscope, uniting in itself the reflecting and refracting elements of that instrument. For, whilst the light from a flame is reflected by two surfaces (the outer concave surface of the concave lens and the internal concave surface of the convex one) into the patient's eye, it is also, on its emergence therefrom, refracted by the effective convex element of the combination, so as to form the usual indirect image of the fundus oculi at the focal length. With such a rough combination I have been able to obtain a distinct image of the optic nerve, retinal vessels, etc.; and I may hence not unreasonably hope a properly constructed mensicus will in itself fulfil the conditions of the mirror and object-lens of an ordinary ophthalmoscope.

I am, etc, J. Z. LAURENCE.

[*British Medical Journal.*

